# ESR Essentials: advanced MR safety in vulnerable patients—practice recommendations by the European Society for Magnetic Resonance in Medicine and Biology

**DOI:** 10.1007/s00330-024-11055-1

**Published:** 2024-09-06

**Authors:** Francesco Santini, Michele Pansini, Xeni Deligianni, Maria Eugenia Caligiuri, Edwin H. G. Oei

**Affiliations:** 1 Basel Muscle MRI, Department of Biomedical Engineering, University of Basel, Basel, Switzerland; 2 Department of Radiology, University Hospital of Basel, Basel, Switzerland; 3 Oxford University Hospitals NHS Foundation Trust, Oxford, UK; 4 Clinica Di Radiologia EOC, Istituto Di Imaging Della Svizzera Italiana (IIMSI), Ente Ospedaliero Cantonale, Lugano, Switzerland; 5 Neuroscience Research Center, Department of Medical and Surgical Sciences, University “Magna Graecia” of Catanzaro, Catanzaro, Italy; 6 Department of Radiology & Nuclear Medicine, Erasmus MC, University Medical Center, Rotterdam, The Netherlands

**Keywords:** Magnetic resonance imaging, Patient safety, Child, Pregnancy, Obesity

## Abstract

**Abstract:**

For every patient, the MR safety evaluation should include the assessment of risks in three key areas, each corresponding to a specific hazard posed by the electromagnetic fields generated by the MR scanner: ferromagnetic attraction and displacement by the static field; stimulation, acoustic noise, and device interaction by the gradient fields; and bulk and focal heating by the radiofrequency field. MR safety guidelines and procedures are typically designed around the “average” patient: adult, responsive, and of typical habitus. For this type of patient, we can safely expect that a detailed history can identify metallic objects inside and outside the body, verbal contact during the scan can detect signs of discomfort from heating or acoustic noise, and safety calculations performed by the scanner can prevent hyperthermia. However, for some less common patient categories, these assumptions do not hold. For instance, patients with larger habitus, febrile patients, or pregnant people are more subject to bulk heating and require more conservative MR protocols, while at the same time presenting challenges during positioning and preparation. Other vulnerable categories are infants, children, and patients unable to communicate, who might require screening for ferromagnetic objects with other imaging modalities or dedicated equipment. This paper will provide guidance to implement appropriate safety margins in the workflow and scanning protocols in various vulnerable patient categories that are sometimes overlooked in basic MR safety guidance documents.

**Clinical relevance statement:**

Special care in the implementation of MR safety procedures is of paramount importance in the handling of patients. While most institutions have streamlined operations in place, some vulnerable patient categories require specific considerations to obtain images of optimal quality while minimizing the risks derived by exposure to the MR environment.

**Key Points:**

- *Patients unable to effectively communicate need to be carefully screened for foreign objects*.
- *Core temperature management is important in specific patient categories*.
- *There are no hard quantitative criteria that make a patient fall into a specific vulnerable category. Protocols and procedures need to be adaptable*.

## Key recommendations

- Attraction of ferromagnetic objects is the highest risk posed by an MR scanner, and it can be mitigated with an accurate patient history. However, this is either impossible or unreliable in some patient groups, such as unconscious or cognitively impaired individuals or infants and children. Resort to the use of other imaging modalities to assess presence of metal, and possibly the usage of ferromagnetic detectors, is recommended when there is the suspicion of an unreliable history. (Evidence level: Low/Moderate—recommendations exist, but no systematic analysis has been done of the specific efficacy of these methods)
- Various patient categories (individuals under sedation or anesthesia, infants) have thermoregulatory issues and need to be monitored with appropriate equipment for hypo- and hyperthermia during MRI. Other categories, such as febrile, obese, or pregnant patients, have lower resistance to hyperthermia (without significantly risking hypothermia). In either case, radiofrequency power deposition should be reduced during an MR examination, by reducing the scanning time and preferring low-specific absorption rate sequences. (Evidence level: Moderate. Effects of radiofrequency (RF) power deposition are well demonstrated through simulations, in vitro, and in vivo)
- These general recommendations do not provide cutoff limits (such as body mass index thresholds) for the execution of specific procedures or scanning protocols. The information provided here offers guidance in the estimation of the risk for the patient classes presented, but the correct diagnostic procedure (i.e., MRI vs an alternative modality, or the scanning protocol) needs to be identified through a case-by-case risk/benefit assessment by the responsible medical professional.

## Introduction

Magnetic Resonance (MR) safety guidelines are a fundamental component of the management and operation of an MR imaging facility. They need to provide a general workflow that ensures the minimization of the risk of MRI examinations for every patient and to streamline operations so that routine can proceed efficiently. However, general guidelines are modeled after an idealized “average” patient, whose characteristics depend on the considered population. Statistical variability in the population makes it inevitable that, occasionally, an individual whose characteristics do not fall within the usual distribution of traits captured in the guidelines needs to be safely scanned. In these cases, a patient-specific evaluation must be performed, and an optimized scanning protocol must be chosen.

In this review, we will present some considerations regarding patient populations that might occur less frequently in standard clinical practice yet who require special attention in ensuring the safety of the MRI exam. Specifically, we will briefly present the inherent risks and mitigation possibilities for patients with fever, who are anesthetized, with larger habitus, pregnant individuals, infants and children, and patients with claustrophobia. Patients with passive and active implants will not be considered in this review, as they are broader themes that require separate articles.

## A brief overview of general MR safety concepts

For every patient or individual that receives an MR scan, or even enters the MR scanner room, the safety risks are posed by the three electromagnetic fields (static B_0_ field, gradient fields, and radiofrequency field) generated by the scanner. These fields directly interact with the body of the person and with all other objects in their vicinity. It is therefore important to understand what these three fields are and how they potentially interact to be able to correctly assess the risk factors for every individual.

### The static field (B_0_)

The static field, which is typically in the order of magnitude of units of Tesla (typically 1.5 or 3 T), is the strongest of the three fields. Unlike the other fields, this field is always present around the scanner. Its primary interactions are with ferromagnetic and paramagnetic objects introduced in the scanner room. The most relevant effect of this field is the attraction force exerted over ferromagnetic materials, which scales with the volume of the material and can reach hundreds of kilograms-force, even for relatively small objects. This force is not mainly dependent on the local magnitude of the magnetic field, but rather on the spatial gradient of the magnetic field, that is, the rate of change of the intensity of the field over a certain distance [1]. This spatial gradient is typically maximum in the immediate proximity of the magnet hardware itself (the edge of the bore) and is zero in the isocenter, where the field is uniform. The secondary effect of the static field is the torque exerted on ferromagnetic objects whose geometry coerces the induced field in a certain direction. This is typically apparent in elongated objects, which, like a compass needle, tend to rotate to align themselves with the direction of the field. Unlike the attraction force, this effect is primarily dependent on the magnitude of the magnetic field and is close to its maximum in the isocenter, where the attraction force is zero. Both these effects are, in general, less of a concern in lower-field systems (below 1.5 T); therefore, scanning at lower field can be an effective way to mitigate the risks arising from B_0_ interactions [2].

### The gradient fields

The gradient fields are magnetic fields whose magnitude varies linearly in space (that is, it is zero in the center, negative on one side of the bore, and positive at the other side), and are switched on and off during the scanning operation. The intensity of these fields is in the order of millitesla/tens of millitesla at their strongest points, which are located at the edge of the bore. They typically switch at a rate of up to a few kilohertz. While their intensity relative to the static magnetic field is not high enough to exert a relevant attraction, they can induce currents that stimulate the peripheral nerves, causing twitching of the muscles, and vibrations in metallic (not necessarily ferromagnetic) objects. They are also responsible for the acoustic noise produced by the scanner, which requires adequate hearing protection [3].

### The radiofrequency field

This field is active only during the scan, and its intensity is in the order of microtesla. Its frequency is in the order of tens of megahertz, and its main effect is the creation of a highly energetic electric field inside the body of the subject. This electric field is proportional to the rate of change of the magnetic field and generates electric currents through the conductive tissues of the body, which are dissipated as heat [4]. This effect primarily results in bulk heating of the body of the subject. The maximum allowed heating under the electrical safety norms published by the International Electrotechnical Commission (IEC) [5] is 0.5 °C (or a resulting body temperature of up to 39 °C) for normal operating mode of the MRI scanner, and 1 °C (body temperature up to 40 °C) for “first-level controlled” operating mode, where increased patient monitoring and, depending on local regulations, medical supervision, is required. Temperatures above those mentioned exceed the certified limits of the scanner and result in “off-label” usage. Clinical MR systems comply with the norm by estimating a quantity called specific absorption rate (SAR). This quantity can be calculated from the sequence parameters, the system hardware, and by a numerical estimation of how the electromagnetic fields are converted into heat by assuming a simplified model of the human body, based on the weight and, sometimes, height of the patient entered during registration [6]. The temporal integration of the SAR provides an estimate of the temperature rise in tissue. From these considerations, we see that SAR is a rather indirect way of controlling the temperature and is based on assumptions about the patient’s body that might not always be correct. SAR is dependent on field strength as it scales quadratically with the Larmor frequency [4], with lower-field systems having a lower power deposition and therefore lower heating when compared to the same sequence executed on a higher-field system. Like for B_0_ interactions, scanning at lower fields can mitigate risks related to heating by achieving lower SAR levels with the same sequence parameters of higher-field systems at the cost of a generally lower image quality.

The secondary effect of the radiofrequency (RF) field is the potential for focal heating when the electric field is focused by the presence of an object that functions as an antenna (such as a conductive cable, implant, or metallic fibers in clothing), or when there is skin-to-skin contact that produces a closed loop, for instance in the lower extremities. The RF magnetic field within the loop causes the induction of currents inside the loop itself, which in turn causes focal heating in the points where current flow is restricted (that is, with high electrical resistance). The former effect can have very serious consequences, especially when occurring inside the body [7], while the latter is generally more benign but rather common, and can result in up to second-degree burns on the skin [8].

## Febrile patients

- Scanning, even in normal mode, can cause a temperature rise of up to 0.5 °C, which can be relevant in febrile patients.
- Allowing heat dissipation and using low-SAR sequences are important measures to avoid hyperthermia.

Fever is a heightened body temperature [9] as a response to various potential causes. It is a common sign in hospitalized patients [10], but a patient with a fever needs to follow stricter MR safety standards, depending on the temperature. If the patient’s core temperature is above 38.5 °C at the start of the scan (possibly even following treatment with antipyretics, or when such treatment is unavailable), even scanning in “normal operating mode” can result in the threshold defined by the IEC norm to be exceeded, and therefore “first-level controlled” care, including potential medical supervision, is required. If the patient’s temperature is above 39 °C, the scanner must be used in normal mode, to avoid exceeding the threshold of 40 °C. Above 39.5 °C, safe scanning cannot be guaranteed by the hardware safeguards, because even normal operating mode might cause the temperature to rise above 40 °C. Scanning above these limits falls directly under the responsibility of the medical professional and might be qualified as “off-label” usage, requiring appropriate risk-benefit assessment which involves estimating the actual patient exposure (based on body part examined, power deposition of the sequences used, hardware setup) and, potentially, management according to local regulations. Apart from the temperature thresholds, efforts should be made to choose protocols with minimal SAR in any febrile patient and to avoid using insulation blankets to allow heat dissipation.

## Anesthetized and unconscious patients

- Anesthetized and unconscious patients might have thermoregulatory issues, and proper temperature monitoring is necessary.
- Screening for ferromagnetic objects, including anesthesia equipment, and MR safety training for non-radiological personnel is required.

Another category of patients that require special care in regulating their body temperature are anesthetized patients. Anesthesia interferes with the natural body’s ability to correctly thermoregulate. While the most common risk is hypothermia, malignant hyperthermia can also rarely occur [11]. In this scenario, and considering the impossibility to communicate with the patient, accurate and responsive temperature monitoring is required to respond to physiological temperature changes and to changes induced by power deposition. Thermocouple temperature monitoring is affected by the MR electromagnetic fields and fiber-optic-based probes, which do not contain metallic parts and are therefore MR safe, should be used instead [12]. The choice of monitoring site is also important and needs to be decided as a tradeoff between accuracy and practicality, accounting for potential delays in the temperature change between core and peripheral locations [13]. Similar considerations might apply to unconscious or otherwise unresponsive patients, if impaired thermoregulation is clinically suspected (e.g., in cases of impaired vasodilation).

Anesthesia equipment is also an obvious concern for ferromagnetic attraction by the scanner, and appropriate MR-conditional equipment must be used inside the scanner room, with all unsafe equipment located outside the attraction area. Proper MR safety training of all personnel involved, including anesthesiologists and nurses, is required to avoid accidents.

## Patients with larger habitus

- Heat dissipation is impaired in patients with larger habitus, and low-SAR scan protocols are preferred.
- Implants in patients with larger habitus might be exposed to higher values of spatial gradient, time-varying gradient intensity and slew rate, and their conditions should be checked against the actual exposure.

Patients with obesity or, generally, a larger habitus, can pose unique challenges in the MR environment. While definitely uncommon, ferromagnetic objects concealed in the patient’s panniculus have been reported in the literature [14]. However, much more relevant risks are posed by RF power deposition and potential incompatibilities of implanted devices.

**Fig. 1:**
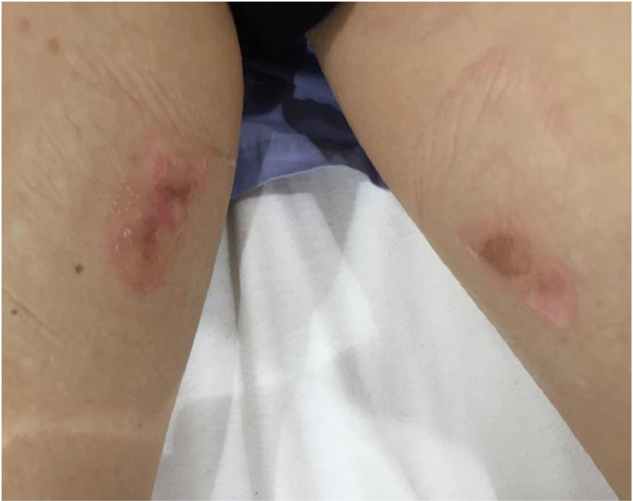
A second-degree burn at the point of skin-to-skin contact of the thighs. Reproduced with permission from Tagell et al [8]

Patients with obesity have a reduced capacity to dissipate heat from RF deposition [15]. Special care must therefore be taken to minimize the SAR, and first-level controlled scanning is not recommended. However, no hard limit in terms of weight or body mass index (BMI) is indicated in the literature as a cutoff for excluding higher-SAR acquisitions, and a risk-benefit evaluation must be performed on a case-by-case basis, maintaining verbal contact with the patient to react to discomfort. Because of the anatomical characteristics of larger habitus, avoiding skin-to-skin or skin-to-bore contact might be more difficult, and this might result in local RF burns (Fig. 1) [8, 16]. Use of appropriate padding to avoid such contact is required.

**Fig. 2:**
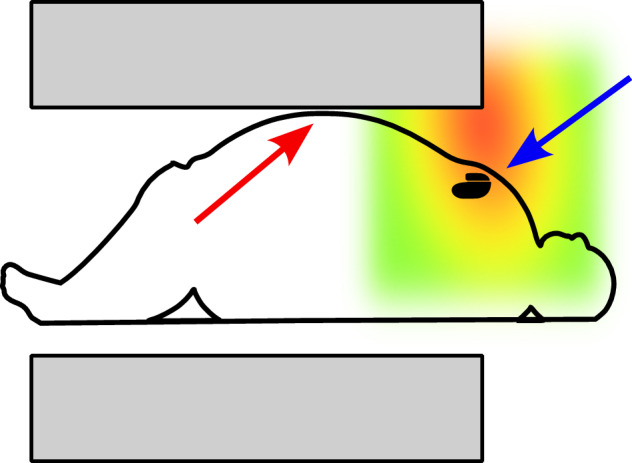
A schematic of a patient with a large habitus in an MR scanner (section of the bore represented in gray). The colored area represents a typical spatial gradient intensity, with red signifying a stronger spatial gradient. An implanted device such as a pacemaker (blue arrow) might find itself in an area of higher spatial gradient with respect to the same device implanted in a person with a smaller habitus. The exact scan conditions need to be checked on a patient-by-patient basis. Contact between the bore and the skin of the patient is also possible (red arrow), and appropriate measures to avoid focal burns must be taken (e.g., insulating pads)

A larger body diameter brings the skin of the patient also closer to the bore surface, where absolute values of gradient fields are higher, and thus, the patient has a higher probability of experiencing peripheral nerve stimulation. This is also relevant when the patient has MR-conditional implants that define exposure limits to spatial gradient or time-varying gradient strengths and slew rates. As the locations of maximum strength for these quantities are located close to the scanner bore, patients with larger habitus might have implants that, when positioned inside the scanner, might be exposed to higher effective values when compared to patients with smaller habitus [17] (Fig. 2).

## Pregnant patients

- MR scanning is safe in any trimester of pregnancy.
- Heat dissipation in the fetus is impaired, and low-SAR procedures need to be selected, well within the limits of normal scanning.
- Contrast agents should be used sparingly due to their prolonged presence in the amniotic fluid.

There is now sufficient evidence supporting that exposure to static magnetic field intensities used in clinical MR is reasonably safe during any trimester of pregnancy [18]. However, RF power deposition can have a heating effect on the fetus, whose only pathway for heat dissipation is via the body of the gestational parent [19]. Simulations show that even by scanning in normal mode, fetal temperature can rise above 38 °C [20]. Contrast agents must also be used sparingly, as there is evidence that they can cross the placental barrier into the fetal circulation, where they are then excreted through the fetal kidney pathway into the amniotic fluid, in which they can remain for a long time [19, 21]. The fetus is generally not considered to be particularly exposed to acoustic noise propagated through the air due to the attenuation provided by the parent’s body [22], but, to prevent transmission of acoustic noise to the ear through direct mechanical coupling with the bed, it should be shielded from vibrations through cushions placed under the patient [19].

## Infants

- Proper fitting of ear protection is necessary.
- Extremely accurate screening for ferromagnetic and conductive material in anything in contact with the infant (clothes, monitoring devices, etc.) is required, as the infant cannot communicate discomfort, and accidents can have particularly severe consequences.

**Fig. 3:**
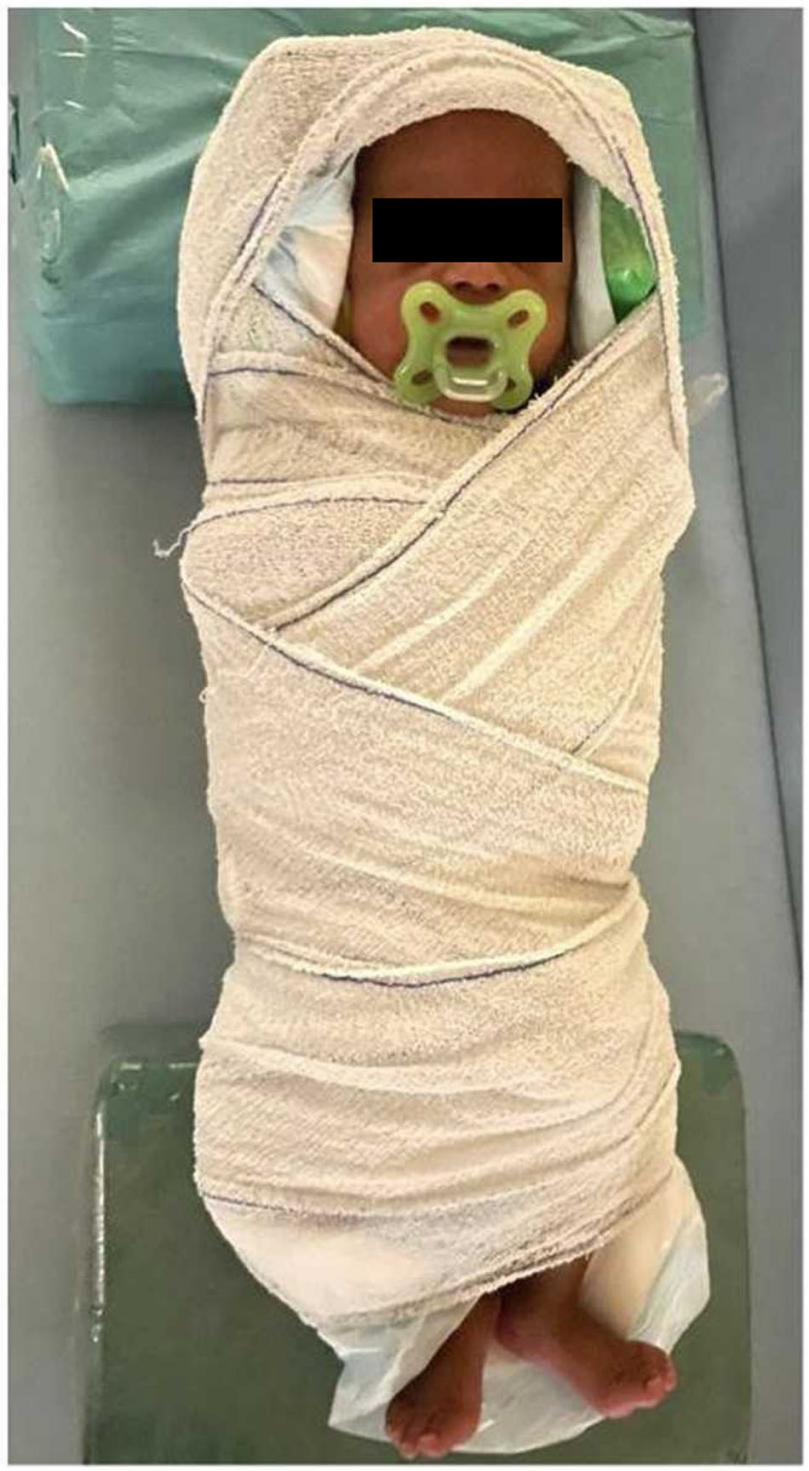
Immobilized infant for MRI. Note how the ear protection equipment is tightly secured on the ears by the wrap. Figure adapted from Korom et al [32], released under a CC-BY license

**Fig. 4:**
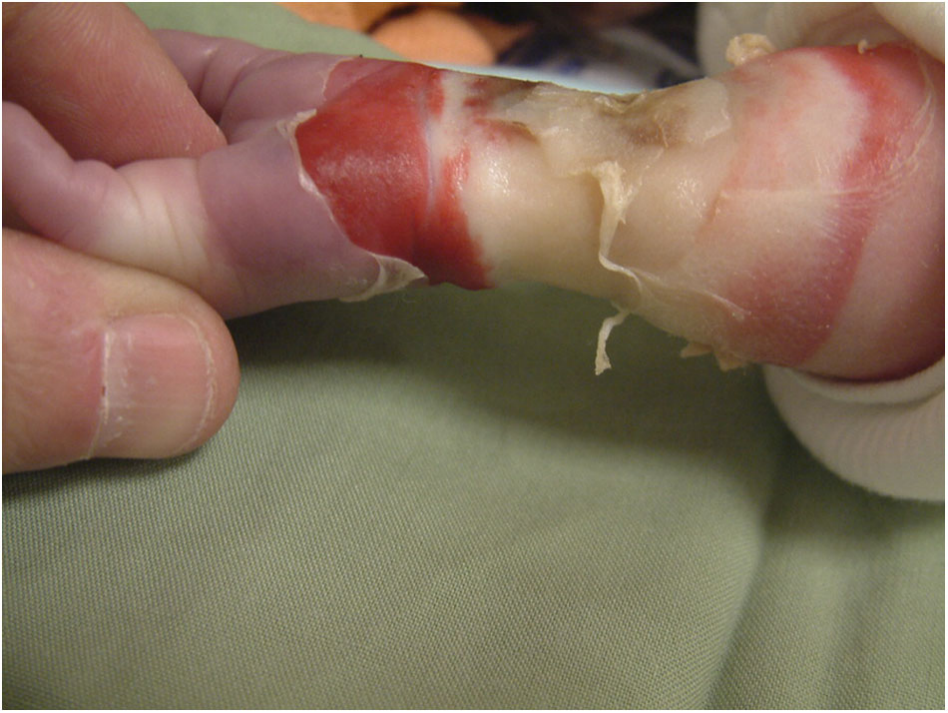
A fourth-degree burn in the forearm of an infant due to contact with a non-MR-conditional pulse oximeter, requiring subsequent amputation. Reproduced with permission from Haik et al [27]

Issues related to the safe scanning of infants and children deserve their own separate treatise [23]. However, we will summarize the main points that need to be taken into consideration for this population. Neonates and infants are unable to communicate and, generally, lie still in the scanner. In order to successfully perform the scan, they usually require sedation and, therefore, correct temperature monitoring, as in the case of anesthetized patients. Considering that preterm and sick babies might naturally have difficulties in thermoregulation, they risk both hypothermia and hyperthermia [24]. However, Malik [25] found that conventional SAR estimations are conservative in neonates, thus providing a safety margin towards hyperthermia. To avoid sedation, immobilization through specialized holding devices is also possible, ideally after feeding (“feed and wrap”, Fig. 3) [26]. However, extreme care must be taken that appropriate ear protection equipment can be worn by the baby, as they are unable to express their specific discomfort. Similarly, all equipment, clothes, and objects in contact with the infant need to be accurately screened for MR safety, as RF burns from the usage of incompatible devices can have vastly more serious consequences when compared to responsive adult patients who are able to request an interruption of the procedure (Fig. 4) [27].

### Children

- Ensure that children have a proper understanding of safety questions, and that they are willing to answer truthfully (if possible, repeat screening in presence and absence of caregivers).

Older children do not generally require fundamentally different precautions than those applicable to adults, as long as they are able to follow instructions and communicate discomfort. However, they might not understand or realize the importance of the screening questionnaire, and parents may be unaware of foreign objects in the child’s clothes, toys, or even in the child’s body. Invisible metallic fibers in clothing, for example, can lead to RF burns [28].

If allowed by local regulations, children should be screened both together and separately from their parents, as they might not be willing to disclose personal information in front of the parent or caregiver (e.g., tattoos or piercings).

## Claustrophobic and autistic patients

- Verbal communication prior to the scan has been shown to mitigate the effects of claustrophobia and improve the outcome of the scans.

Approximately 1.2% of all patients undergoing an MR investigation suffer from claustrophobia [29]. While this does not pose a direct safety risk in itself, the discomfort of the patients can lead to impaired image quality and make it impossible to perform the scan, with direct consequences on the management of the patient. Verbal communication prior to and during the scan has been shown to mitigate the effects of claustrophobia and improve scan outcomes [30]. In some cases, sedation is required. If sedation is necessary, patient monitoring appropriate to the level of sedation and the possibility of interaction between the patient and the personnel must be implemented. This includes a particularly accurate screening for potential sources of RF burns (skin-to-skin and skin-to-metal contact), ear protection, and temperature monitoring if deemed appropriate. Similar considerations apply to autistic individuals, for whom psychological discomfort can be a primary cause for a failed exam. As with claustrophobic patients, communication with autistic patients, familiarization with the scanner environment, reduction of scanner noise, and distraction techniques can avoid sedation. A comprehensive review of the management of autistic patients is given by Stogiannos et al [31].

## Summary statement

Every patient has a unique set of risk factors associated with an MR scan, and the evaluation of the appropriate course of action needs to be performed by weighing them against the potential benefits. The risks associated with every patient are always arising from the interaction of the patient’s body, and of foreign objects, with the three electromagnetic fields generated by the scanner: the static field, the gradient fields, and the RF field.

**Fig. 5:**
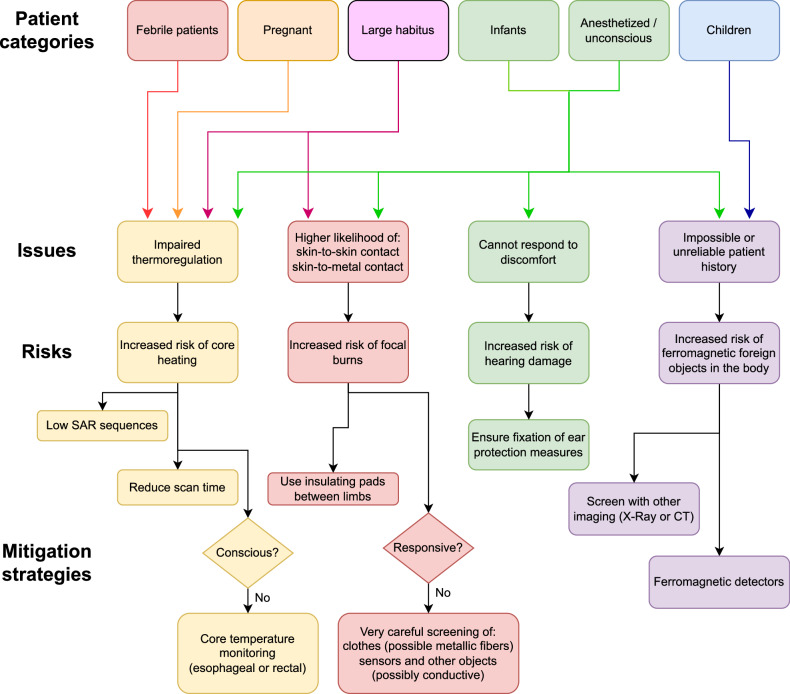
Flowchart representing the main risks and the main mitigation strategies for the categories of patients considered

When a patient belongs to a certain category, the risks associated with one or more of these fields are increased (see the flowchart in Fig. 5).

As a broad classification, patients for whom a reliable history cannot be obtained, such as children, unconscious, or cognitively impaired patients, are at a heightened risk of having foreign objects that could be displaced or heated during the scan and need to be carefully screened. Patients with difficulties in thermoregulation, such as unconscious, febrile, obese, or pregnant patients, need to be exposed to more conservative levels of RF power to avoid hyperthermia and monitored if appropriate. Patients who cannot communicate during the scan, such as infants or unconscious individuals, need to be additionally protected against acoustic noise.

## Patient summary

Magnetic resonance imaging is a safe procedure when performed within the appropriate safety boundaries, however it is not free of potential risks. In some patient categories, which are less commonly encountered in daily practices, these risk factors are heightened, and special care needs to be taken when evaluating the correct procedure to perform, to minimize the risks and maximize the benefit to the patient.

## Abbreviations

IEC: International Electrotechnical Commission
RF: Radiofrequency
SAR: Specific absorption rate
